# Efficacy of an indicated prevention strategy on sickness absence and termination of the employment contract: a 5-year follow-up study

**DOI:** 10.5271/sjweh.3945

**Published:** 2021-04-27

**Authors:** Sophie H Klasen, Ludovic GPM van Amelsvoort, Nicole WH Jansen, Jos JM Slangen, Gladys Tjin A Ton, IJmert Kant

**Affiliations:** CAPHRI School for Public Health and Primary Care, Department of Epidemiology, Faculty of Health, Medicine and Life Sciences, Maastricht University, Maastricht, The Netherlands; Occupational Health Service Beter, Amsterdam, The Netherlands

**Keywords:** preventive intervention, RCT, sick leave

## Abstract

**Objective:**

It was shown that an indicated prevention strategy (IPS), based on screening and early intervention, can considerably decrease future risk of long-term sickness absence (LTSA>28 days) over one year. Given the nature of the interventions, the potential of an effect extending beyond the original one year of follow-up might be present. This study aims to determine the efficacy of this IPS on LTSA and termination of employment contract over five years by extended follow up of IPS trials.

**Methods:**

Company records on sickness absence and termination of employment contract over five years were used from two randomized controlled trials (RCT) on the efficacy of the IPS (RCT I employees at high-risk for LTSA: intervention: N=263; RCT II high-risk employees with concurrent mild depressive complaints: intervention: N=139). Survival analysis was used to model time until the first LTSA episode and termination of employment contract.

**Results:**

RCT I showed a decrease of 43.2 days of sickness absence (P=0.05) and a lower 5-year risk of LTSA in the intervention, as compared to the control group [hazard ratio (HR) 0.61, 95% confidence interval (CI) 0.41–0.90], however no considerable impact on employment contract (HR 0.85, 95% CI 0.54–1.35) (intention-to-treat, ITT). For RCT II, we found no large difference in days of SA and no difference in LTSA risk over five years (HR 1.31, 95% CI 0.70–2.47), whereas the risk of termination of the employment contract was lower (HR 0.62, 95% CI 0.39–0.99) (ITT).

**Conclusion:**

Effects of the IPS were observed over five years, albeit differential between the two approaches. A combination of elements of both interventions might lead to optimal results but needs further study.

Long-term sickness absence (LTSA) has large consequences in terms of health and costs for employees, employers, and society (1–3). LTSA is seen as a precursor of permanent work disability, early retirement due to ill health, and even mortality (3, 4). Many studies have shown that returning to work after a period of LTSA remains very difficult and may even result in financial difficulties over time due to unemployment (1, 2, 5, 6). Perceived poor health, mental health issues, or chronic conditions are known factors that could determine the termination of the employment contract with the company, as a result of disability pensions or unemployment (7). Therefore, preventing LTSA may be positively associated with fewer employees having to exit employment due to ill health. In The Netherlands, termination of the employment contract can be related to disability, retirement, job loss, or voluntary leave. Preventing LTSA is of utmost importance and may result in improving the health of employees, fewer costs due to a decrease in days of sickness absence (SA), and the prevention of work disability (3).

Musculoskeletal disorders and stress-related ill health are seen as the most important reasons for LTSA (8, 9). However, the etiology of SA is often multifactorial which makes it difficult to comprehend and requires a holistic understanding (10–12). Many factors have been associated with an increased LTSA risk, for example, age, gender, lifestyle, poor health, SA records, physical workload, and psychosocial working conditions (10, 13–15). Therefore, individual or indicated prevention might result in better outcomes since it focuses on a broad range of potentially interrelated factors, in contrast to population or general prevention, which is often restricted to one or two factors. Essential is here the focus on treating individuals who are at risk of reporting sick in the future but are not yet currently on sick leave.

Two prerequisites for a successful indicated prevention strategy (IPS) to prevent LTSA are the ability to (i) detect individuals who are at high risk for future LTSA and (ii) provide these individuals with effective treatment at an early stage. A strategy meeting both prerequisites has shown its efficacy in two Dutch randomized controlled trials (RCT) (16, 17). While the RCT differed in study population and type of early intervention, both used screening and structured early intervention (16–18). Earlier studies have shown the efficacy of this prevention strategy in reducing days of SA over a 12-month interval (16, 17). Furthermore, a recent meta-analysis (19) showed that other interventions based on the principles of IPS could have considerable effects on SA. Duijts et al (20) reported 15.5 compared to 18.8 SA days [hazard ratio (HR) of -0.15, 95% confidence interval (CI) -0.23–-0.07], Lerner et al (21) showed 7.1% improvement in productivity due to less SA (P<0.01) and 29.5 compared to 26.0 effective weekly hours (P=0.008) and, over a period of one year, Taimela et al (22) showed a mean difference of 11 days between intervention and control group in favor of the intervention group (20–22). The results from comparable IPS only showed short-term effects (4–24 months), all of which were comparable to the average one-year results for RCT I=12.1 days and RCT II=23.3 days.

However, the long-term efficacy of an IPS has not yet been studied in terms of SA. While the efficacy in terms of decreasing SA during one year of follow-up was large in RCT I, this could indicate that the intervention has lasting effects on help seeking behavior, which could possibly decrease SA over a long time period (23–25). Especially since one might assume that early contact with the occupational physician (OP) could result in sustainable work adjustments or other improvements in working conditions. With regards to the preventive intervention used in RCT II, which was based on Problem Solving Therapy (PST) and Cognitive behavioral Therapy (CBT), it was found that, over one year, SA as well as depressive complaints decreased (17). However, given the aim of the intervention (ie, to enhance coping ability), long-lasting effects beyond the reported one year might also be expected for this intervention. Nonetheless, so far, long-lasting effects have not yet been described for this or similar interventions, as apparent from a recent meta-analysis (19). The expectation of long-lasting effects comes from studies on the effect of CBT in terms of depressive complaints, which suggests that CBT might have enduring effects that extend beyond the end of treatment, supporting our hypothesis that the intervention from RCT II could also lead to a sustainable decrease in SA at 5-years follow-up (26–31). Demonstrated long-term efficacy is highly relevant for social and economic reasons since LTSA often is associated with high costs (2). Therefore, this study aimed to examine the efficacy of an indicated prevention strategy to prevent LTSA, through record linkage of the RCT participant’s data on SA parameters and termination of employment over a 5-year follow-up period.

## Methods

### Design, procedure and participants

Two RCT were conducted among office workers who were classified as high risk for future LTSA by a screening questionnaire called the ‘Balansmeter’ in Dutch. The current paper describes a follow-up study on indicators of labor participation with a focus on SA parameters and termination of the employment contract. Similarities can be found between the preventive interventions in RCT I and RCT II in the timing of the preventive intervention and the use of a screening instrument to classify employees as high risk for LTSA. However, the preventive interventions differ in the type and intensity of treatment.

### Screening instrument

The screening instrument (Balansmeter) was developed to identify employees at high risk for future LTSA in an office environment before they report sick. The Balansmeter was internally validated on data of the Maastricht Cohort study and externally validated on a large sample of employees from the same company in which both RCT were conducted (18, 32). Detailed information about the screening instrument can be found in the supplementary material (www.sjweh.fi/show_abstract.php?abstract_id=3945).

### RCT I

Starting in 2003, RCT I invited 9863 employees to participate in the study, of which 4950 responded to the questionnaire. Employees were selected if they scored above the cut-off point of the Balansmeter, which indicated that they were at high risk for future LTSA. Exclusion criteria were employees (i) already on sick leave, (ii) receiving OP care at the time of completing the screening questionnaire, (iii) who left the company during the RCT period, and (iv) who were pregnant. This resulted in N=263 employees eligible for allocation in the intervention or control groups. A detailed description of the selection procedure of participants is described elsewhere (16). The original follow-up period for RCT I was one year, extended to five for the current study. The allocation of participants in RCT I is shown in supplementary figure S1. The number of study participants decreased over time due to the departure of employees from the company because of disability, retirement, job loss, or voluntary leave.

For RCT I, employees in the intervention group received a structured early consultation by the OP/OHP, which may already be viewed as a short intervention due to the time involvement, often followed by further consultations within the occupational health service. The consultation was held according to a protocol consisting of different steps, in which the main symptoms were discussed and the relation between their symptoms and the risk for future LTSA explained. Finally, the expectations and benefits of early treatment were discussed with the employee. The consultation could then be followed by a targeted intervention to focus directly on the identified issues. Different interventions were applied (eg, psychological interventions, lifestyle interventions, and interventions by company counselors). This resulted in 84 employees having a consult with the OP of which 14 received additional treatment, as retrieved from questionnaires completed by the OP (16). The focus of this IPS is the early timing – before SA occurs – rather than the type of intervention. The control group received care as usual (ie, when there was a need). A detailed overview of this preventive intervention can be found at Kant et al (16).

### RCT II

The selection process for RCT II started in 2007, with 9157 employees responding to the study invitation. Employees were eligible if they were classified as being at high risk for future LTSA and additionally had mild depressive complaints. Depressive complaints were assessed using the depression subscale of the Hospital Anxiety and Depression Scale (HAD-D) which consists of 7 items ranging from 0–21 (33). The employees were classified as having mild depressive complaints when they scored ≥8 points on the HAD-D. Exclusion criteria were: fully or partly absent from work, already receiving treatment by the psychologist/psychiatrist at the time of completing the screening questionnaire, pregnant or on maternity leave. This resulted in N=139 employees who were eligible for randomization in the intervention or control groups. Lexis et al (17) described RCT II in detail.

The original follow-up period for RCT II was 12 months, extended to five years for the current study. The number of study participants decreased over time due to termination of the employment contract as a result of pension, disability benefits, voluntary leave or, involuntary leave. A flow diagram of study participants is shown in supplementary figure S2.

Employees in the intervention group received a psychological treatment based on principles of PST and CBT to enhance their coping ability to prevent LTSA and stimulate personal well-being. Seven individual sessions of 45 minutes each were provided. After each session, homework assignments were given to the employees and discussed in the following session. The number of sessions could be extended to 13 sessions if needed. Ten psychologists conducted the sessions and received a 2-day training session before the intervention and a 1-day booster session during the study (17). The focus of this IPS is the early timing – before SA occurs – as well as the intensity of the individual sessions. Employees in the control group received care a usual.

### Outcome measures

#### Primary outcome

Indicators of labor participation were investigated by SA parameters, which entailed the mean duration of SA (including >28 SA days), SA frequency, the percentage of LTSA (>28 days SA), and the time until the first onset of LTSA. The percentage of LTSA was calculated for each year separately even if the period of LTSA has started the previous year. The occupational health service from a financial service provider ‘Beter’, provided us with SA data through record linkage on an individual level with company sick-leave registries and anonymized according to the current General Data Protection Regulation. SA duration was measured in both RCT in calendar days according to the defined time window: 1–5 years of follow-up.

#### Secondary outcome

Termination of the employment contract was characterized by the time (in months) until an employee departed the company during the follow-up period. Termination of employment could be due to disability, retirement, job loss, or voluntary leave. The HR office from the company under study provided us with the termination of employment contract dates. Especially the relation between IPS for RCT II employees might be of interest, while work disability studies have shown a strong relation with coping abilities and return to work behavior (30, 35). Termination of the employment contract was perceived to be important, especially for RCT II employees, where the preventive intervention was developed to improve their coping abilities. Further it was investigated if SA and LTSA were precursors for the time until termination of the employment contract.

### Statistical analysis

The indicators of labor participation were analyzed according to the intention-to-treat (ITT) principle. Per protocol analyses are provided in the supplementary material. Poisson regression was used to estimate the efficacy of the IPS in terms of the mean duration of SA, SA frequency, and the percentage of LTSA. The time until the first onset of LTSA and the time until termination of the employment contract was examined with multivariate Cox regression analyses. All analyses were conducted for RCT I and RCT II separately. The analyses were adjusted for the following covariates: age, gender, job function/education level (available data differed between RCT I and RCT II), and long-term illness previous to the screening questionnaire. These covariates were chosen due to their important predicting ability for SA (36, 37). A Chi-square test was used to investigate if SA or LTSA were precursors for the departure of employees from the company. All analyses were conducted per year for five years of follow-up, except for the multivariate Cox regression which was estimated for five years of follow-up. All analyses were conducted with the use of SPSS version 25 (IBM Corp, Armonk, NY, USA).

## Results

Baseline characteristics of the participants from RCT I and II are displayed in table 1. Age, mean number of years working for the company and working hours per week were similar for the control and intervention groups. Small differences were apparent with regards to gender, educational level, and long-term illness.

**Table 1:** Descriptive characteristics of study population randomized controlled trials (RCT) I and II.[SD=standard deviation.]

Variables	RCT I				RCT II			
	
	Control (N=131)		Intervention (N=132)		Control (N=70)		Intervention (N=69)	
			
	N (%)	Mean (SD)	N (%)	Mean (SD)	N (%)	Mean (SD)	N (%)	Mean (SD)
Gender (male)	90 (68.7)		97 (73.5)		43 (61.4)		42 (60.9)	
Age (18–65 years)		46.6 (8.3)		46.3 (8.4)		47.1 (9.5)		48.4 (8.7)
Highest level of education/job function ^a^								
Low	65 (49.6)		53 (40.2)		6 (8.9)		5 (7.9)	
Medium	46 (34.9)		63 (47.7)		45 (67.3)		49 (77.8)	
High	20 (15.5)		15 (11.5)		16 (23.9)		9 (14.3)	
Working hours/week		33.9 (4.8)		34.4 (3.9)		34.9 (4.9)		34.8 (4.5)
Years working for the company		23.5 (11.1)		23.9 (11.0)		n.a.		n.a.
Long-term illness	(47.5)		(54.5)		(51.5)		(59.1)	

a Does not add up due to missing data.

### Results sickness absence parameters and termination of employment contract (RCT I)

Table 2 presents the results according to the ITT principle for RCT I. On average, the intervention group had fewer mean days of SA in each year compared to the control group. Mean days of SA differed only borderline statistically significant for the five years of follow-up with a difference of 43.2 days of SA between the control and intervention groups. The Per Protocol analysis showed statistically significant differences between the intervention and control group for each year except four years of follow-up. After five years a difference of 52.8 mean days of SA (95% CI 3.21–123.31) between the control and intervention groups in favor of the latter was found. Results from the PP analysis are available in the supplementary material (table S1 and figures S1 and S2). The percentage of LTSA was lower in the intervention compared to control group and was statistically significant after three, four, and five years of follow-up according to both ITT and PP analysis.

**Table 2:** Overview efficacy of the indicated prevention strategy on sickness absence parameters for randomized controlled trial I (intention to treat analyses). [LTSA=long-term sickness absence; SD=standard deviation.]

Follow-up period	Control group				Intervention group				Difference		P-value ^a^	P-value ^b^
		
	Mean (SD)	%	Median	N	Mean (SD)	%	Median	N	Mean	%		
2-years												
Total SA duration ^c^	68.4 (88.1)		35.8	114	55.5 (76.4)		24.0	125	12.9		0.164	0.08
SA frequency	5.42 (5.47)		4.0	114	4.85 (3.83)		4.0	125	0.57		0.297	0.089
Percentage of LTSA ^d^		26.7		35		23.5		31		3.2	0.306	0.225
3-years												
Total SA duration ^c^	104.3 (119.8)		56.6	109	82.2 (105.9)		36.02	119	22.1		0.099	0.069
SA frequency	7.66 (7.38)		6.0	109	6.89 (5.30)		6.0	119	0.77		0.313	0.078
Percentage of LTSA ^d^		36.6		48		24.2		32		12.4	0.011	0.005
4-years												
Total SA duration ^c^	125.2 (150.3)		65.0	99	112.5 (150.5)		49.5	114	12.7		0.485	0.376
SA frequency	9.36 (9.44)		7.0	99	8.62 (6.36)		7.0	114	0.74		0.450	0.091
Percentage of LTSA ^d^		35.1		46		27.3		36		7.8	0.042	0.025
5-years												
Total SA duration ^c^	166.3 (202.7)		93.5	94	123.1 (167.1)		65.0	101	43.2		0.06	0.055
SA frequency	11.44 (11.18)		9.0	94	10.64 (7.59)		9.0	101	0.80		0.522	0.135
Percentage of LTSA ^d^		35.9		47		24.2		32		11.7	0.019	0.007

a Crude analysis using Poisson regression without adjustments for covariates.

b Adjusted analysis using Poisson regression for covariates: age, gender, job function and long-term illness.

c Total SA duration including >28 SA days.

d Percentage LTSA is calculated annually.

The course over time until the first onset of LTSA for the intervention and control groups is shown for RCT I in figure 1A. According to this survival curve of figure 1A, after five years, 35% of the intervention group was on sick leave for >28 days as compared to 50% of the control group. For the intervention group, this resulted in an average time until the first onset of LTSA of 42.3 months compared to 36.1 months for the control group. According to the ITT principle, adjusted for the covariates age, gender, job function, and long-term illness this gave a HR of 0.61 (95% CI 0.41–0.90).

**Figure 1A:**
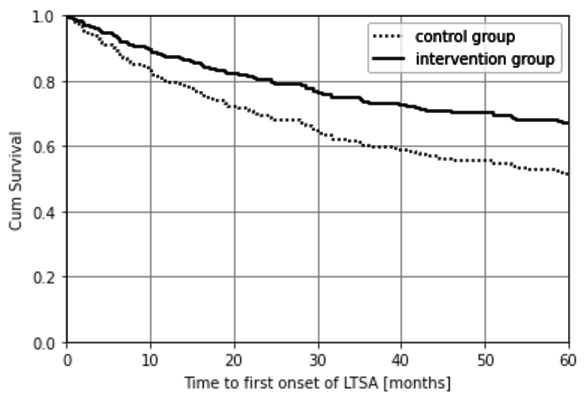
Time till first spell of LTSA (RCT I) according to the intention to treat principle (HR 0.61; 95% CI 0.41–0.90).

The average time until termination of the employment contract was 53.8 and 51.8 months for the intervention and control groups, respectively, HR 0.85 (95% CI 0.54–1.35) (adjusted, figure 1B). Employees who left the company during the five years’ follow-up did not differ statistically significant in terms of days of SA or LTSA from those who did not leave the company.

**Figure 1B:**
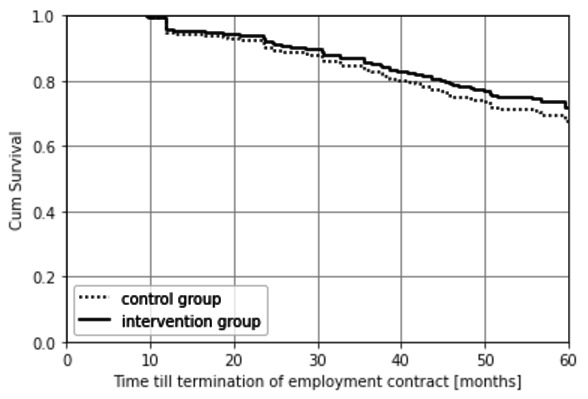
Time till exit from the company contract in months (RCT I) according to the intention to treat principle (HR 0.85; 95% CI 0.54–1.35).

### Results sickness absence parameters and termination of employment contract (RCT II)

The SA results for RCT II according to the ITT are presented in table 3. No differences were found in SA duration and frequency between the intervention and control group according to the ITT and PP analysis. Cumulative after 3–5 years, the control group had less SA days compared to the intervention group, however this evidence is very uncertain and not statistically significant according to both ITT and PP analysis. Results from the PP analysis are available in the supplementary material (table S2 and figures S3 and S4).

**Table 3:** Overview efficacy of the indicated prevention strategy on sickness absence parameters for randomized controlled trial I (intention to treat analyses). [LTSA=long-term sickness absence; SD=standard deviation.]

Follow-up period	Control group				Intervention group				Difference		P-value ^a^	P-value ^b^
		
	Mean (SD)	%	Median	N	Mean (SD)	%	Median	N	Mean	%		
2-years												
Total SA duration ^c^	81.2 (114.2)		29.5	56	71.1 (109.0)		29.0	65	10.1		0.555	0.554
SA frequency	3.68 (2.88		3.0	56	3.68 (2.83)		3.0	65	0		0.997	0.748
Percentage of LTSA ^d^		18.6		13		21.7		15		-3.1	0.985	0.975
3-years												
Total SA duration ^c^	106.6 (135.8)		43.5	50	110.5 (143.0)		57.0	60	-3.9		0.868	0.996
SA frequency	5.46 (4.04)		4.5	50	5.47 (4.39)		4.0	60	-0.01		0.993	0.587
Percentage of LTSA ^d^		21.4		15		30.4		21		-9	0.597	0.695
4-years												
Total SA duration ^c^	133.0 (185.2)		36.5	40	145.7 (185.1)		74.8	58	-12.7		0.731	0.891
SA frequency	6.45 (5.42)		5.0	40	6.76 (5.07)		5.5	58	-0.31		0.758	0.930
Percentage of LTSA ^d^		17.1		12	30.4	21		-13.3	0.547	0.746
5-years												
Total SA duration ^c^	170.5 (249.0)		54.1	33	197.6 (251.3)		98.0	47	-27.05		0.596	0.776
SA frequency	7.15 (5.97)		6.0	33	8.08 (5.83)		7.0	47	-0.93		0.459	0.777
Percentage of LTSA ^d^		17.1		12		29.0		20		-11.9	0.619	0.786

a Crude analysis using Poisson regression without adjustments for covariates.

b Adjusted analysis using Poisson regression for covariates; age, gender, education level and long-term illness.

c Total SA duration including >28 SA days.

d Percentage LTSA is calculated annually.

The average time until the first onset of LTSA was 38.1 and 33.1 months for the intervention and control groups, respectively, as shown in figure 2A. However, this difference was not statistically significant given the adjusted HR 1.31 (95% CI 0.70–2.47).

**Figure 2A:**
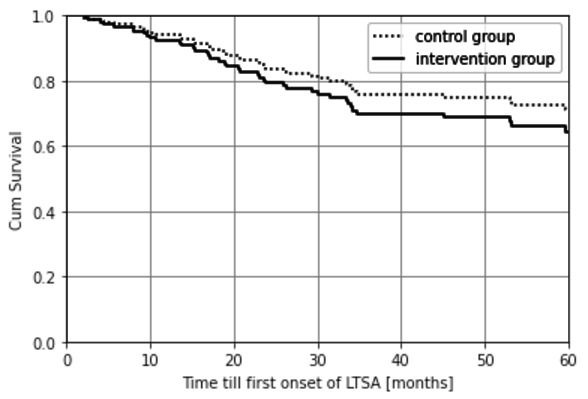
Time till first onset of LTSA (RCT II intention to treat principle) (HR 1.31, 95% CI 0.70–2.47).

The average time until termination of the employment contract for the intervention group was 48.7 months and for the control group 40.2 months. As shown in figure 2B, according to the ITT principle, after five years 45% of the intervention group as compared to 62% of the control group departed the company. The difference between the groups was significant with a HR 0.62 (95% CI 0.39–0.99) (adjusted). According to the PP analysis, 35% of the intervention group departed from the company as compared to 60% from the control group with a HR 0.48 (95% CI 0.27–0.85) (adjusted, see supplementary figure S4). The employees who departed the company did not differ statistically significant from the employees who stayed with the company during the five years in terms of SA and LTSA.

**Figure 2B:**
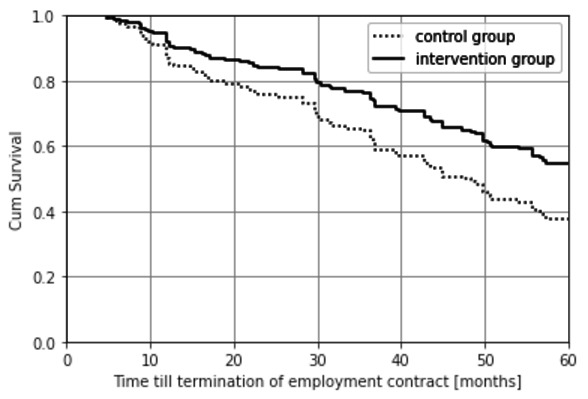
Time till termination of the employment contract with the company in months (RCT II intention to treat) (HR 0.62, 95% CI 0.39–0.99).

## Discussion

The study aimed to estimate the efficacy of an IPS on the prevention of LTSA and termination of the employment contract over five years of follow-up. This was a follow-up study on employees from two Dutch RCT with an original follow-up of 12 months. The focus was on employees classified by a screening questionnaire as being at high risk for future LTSA. Additionally, RCT II only included employees with mild depressive complaints. The RCT also differed in type and treatment intensity. Previous results from 12 months’ follow-up showed statistically significant reductions in days of SA in favor of the intervention group (RCT I and RCT II), as well as a reduction of depressive symptoms in the intervention group (RCT II).

This study showed over five years that, on average, the intervention group (RCT I) had 43.1 fewer days of SA compared to the control group (P=0.05) and showed significantly less LTSA (24.2% versus 35.9%; P=0.019). The intervention group had a significantly longer time until their first period of LTSA (42.3 versus 36.1 months) (HR 0.61, 95% CI 0.41–0.90). Termination of the employment contract did not differ between the control and intervention group. For RCT II participants, no significant differences in days of SA and LTSA were found between the groups after 12 months of follow-up. The time until the first onset of LTSA was somewhat longer in the intervention compared to control group over five years (HR 1.31, 95% CI 0.70–2.47). Whereas, the risk of termination of the employment contract was significantly lower in the intervention group (HR 0.62, 95% CI 0.39–0.99).

Only six other studies to date reported results of an IPS on SA or work-related outcomes (20–22, 38–40). However, none of these reported outcomes beyond 24 months of follow-up, and five of the six only covered at most one year follow-up. This means that beyond the two RCT described here, comparison with other results from studies describing long-term effects (five years) is not possible. The comparison of our study results with other preventive approaches aimed at SA, not based on indicated prevention, is also challenging as the majority of these studies either focused on the general population or employees already on sick leave and showed different results (41–43). The 5-year follow-up of our study with regards to the efficacy of LTSA or work disability interventions seems rather unique, as even with a general focus of studies on SA reduction, no other studies were found for comparison.

The 5-year study period was chosen due to the expected long-term effects as potential changes in help-seeking behavior might occur among the employees at high-risk for future SA and subsequently could lower the threshold to visit a physician, especially visits to an OP, who can also advise in adjustments to the work situation or stimulate addressing potential issues with a supervisor, management or colleagues (23). The healthcare usage is currently being analyzed and the preliminary results show that the IPS for both RCT increases short-term healthcare usage (Klasen et al, unpublished). Regrettably, due to privacy reasons, no further information could be retrieved on the content and number of consults with the OP and the decisions made. Therefore, one can only hypothesize that the early consult with the OP led to a long-lasting decrease in SA due to the early awareness of a health problem/personal issue. But also the process of problem identification and the drafting of a concrete plan of action by the OP might have contributed in long lasting effects as reported. However, further studies should ideally verify these results in similar and other study populations.

The results of RCT II were unexpected, as no decrease in SA was observed during 2–5 years follow-up. Due to the long-term results from CBT regarding depressive complaints, as reported in several long-term studies (covering 3–6 years), one might expect that a more lasting effect would exist also for SA (28–30). However, in the current study, no evidence was found for a sustainable decrease in SA after one year. It might be that the number of subjects was insufficient to find potential small effects. Additionally, the difference in efficacy of long-term effects of CBT interventions might be the result of more severe depressive complaints as compared to the less severe ones of the current study participants (29). Furthermore, the current study did not include booster sessions during the prolonged follow-up, which is expected to have led to better outcomes (28). Further studies might focus more on the sustainable spectrum of effects on the early consultation with the OP and the PST/CBT intervention and investigate the efficacy elements of each strategy.

The efficacy differences between the RCT might at least partially be due to the different selection criteria of RCT participants. In addition to being at high risk for future SA, RCT II participants were selected for experiencing mild depressive complaints. Therefore, other factors might be of importance for an approach to prevent future SA compared to the general high-risk population. Depressive complaints might give rise to, eg, stigmatization, lower socioeconomic status, loss of a valuable source of social support (44, 45). Possibly, more holistic care is needed for these people, while their healthcare needs will be larger, due to different health and personal influences as a result of their illness. A difference in efficacy may also be explained by different intervention characteristics. RCT I focused on issues that emerged from the early consultation with a suited intervention, while RCT II was based on a psychological treatment with principles of PST/CBT and was developed to improve employees coping skills. Moreover, the intervention in RCT II involved many different sessions and was, therefore, more intense than RCT I.

This is the first study, as far as we know which investigated the termination of the employment contract in an indicated preventive setting. In general preventive interventions, it was often studied as work disability or workability (46, 47). The termination of employment contract in this study is less specific, it could be due to disability, retirement, job loss, or voluntary leave and is therefore difficult to compare to other studies. Although in RCT II the risk of termination of employment was found to be substantially lower in favor of the intervention group, we do not have a clear explanation for this positive and relevant effect. Possibly, PST/CBT makes the intervention group more resilient and more proactive with regards to solving potential participation problems.

The strengths of this study are its randomized and longitudinal design, objective measurement of SA, and termination of the employment contract, data availability of two RCT, and no large differences in the ITT and PP outcomes within the RCT were observed. The termination of the employment contract dates provided by the company is expected to be the golden standard (48). There was no differential loss to follow-up, which resulted in an even comparison over the years. Employees who left the company did not differ in terms of SA and LTSA compared to those who stayed and, therefore, we expect it did not have a large impact on the average days of SA per year. As one might assume that censored employees have similar prospects of reaching the outcome as those who continued to be followed, bias of the survival analysis due to right-censoring is assumed to be low. The researcher who analyzed the data was blinded due to anonymized personnel numbers. Contamination in the first three years of study was not observed while the control group did not receive an early OP consultation. Possibly other healthcare or interventions were used but, in the strict sense of the early consultation as being an essential part of the intervention, these were not seen as co-interventions.

It is conceivable that contamination between the intervention and control groups occurred as both groups received the IPS according to the protocol of the first RCT after three years (if identified as high risk yet again). Unfortunately, we were not able to retrieve if, and how often, this occurred. This might have reduced the contrast between the groups and most likely this bias has led to an underestimation of the efficacy, assuming the intervention is effective.

The underlying reason for the termination of the employment contract was unavailable to the researchers due to the EU’s strict General Data Protection Regulation. No distinction could be made between the termination of the employment contract due to disability, retirement, job loss or, voluntary leave. Therefore, the results from this study may be seen as the first step towards better understanding the efficacy of an IPS on termination of the employment contract, and future studies may further distinguish the reasons for leaving the company.

In addition, a technical malfunction in the data merge resulted in a loss to follow-up in the first year, of N=14 for RCT I and N=10 for RCT II. There were no indications that this loss to follow-up was selective and unlikely to be related to the outcome or the interventions and, therefore, would not have biased the results. The trials were carried out in a large company in The Netherlands in the context of its specific social system, and RCT participants were office workers with access to an occupational health service with a very high service level. Although such an indicated preventive approach could be effective in many countries and contexts, extrapolation of the reported results should take the national and labor context into account while SA and its prevention is highly dependent on cultural as well as legislative factors (3). Thus, adaptation should be done with care, tailoring and testing of the screening and interventions under study. In companies with a lower occupational health care service level, the contrast between intervention and control groups may be different, resulting in higher or lower effect sizes.

For future studies, the first step should be to validate the results from this study in a different study population. Possibly, with additional data gathering concerning healthcare usage or workplace involvement to better understand the effective elements of both interventions. Moreover, future studies should extend their follow-up period to investigate the full potential of their intervention while currently, similar studies often focused on a short period. Furthermore, it would be interesting to investigate if IPS differs in efficacy for mental or physical health complaints and leads to medicalization. The IPS is focused on individual health/general problems and the company was only involved when the employees wanted to express their issues. However, the problems might be related to the organization itself, and possibly involving the organization could improve the shared responsibility for employees’ health. Especially the multifactorial factors of LTSA give a lead to a more holistic approach. However, this could encounter inherent difficulties due to privacy issues. Future studies should investigate if a holistic IPS is feasible in an organization where the focus remains on individual treatment, valuing the doctor/patient relationship and privacy issues.

The screening interval of three years of this IPS seems suited to create long-term effects while especially the efficacy of LTSA is visible after two years. However, the screening interval period has not yet been validated, and it would be interesting for future studies to investigate what is acceptable in terms of costs and benefits for both employees and employers.

To conclude, after one year, the IPS resulted in a large reduction in days of SA. With an extended follow-up of five years, this strategy showed a reduction in days of SA and LTSA for the intervention compared to control group (in RCT I). However, this decrease was not found for participants with mild depressive complaints after the first year (RCT II). For participants receiving a psychological treatment based on PST/CBT, RCT II showed that the intervention had a positive effect on preventing termination of the employment contract. This relation was not found in RCT I. A different type of intervention and study population might have resulted in different results for the RCT. Thus, the best elements of both interventions should be further studied.

## Supplementary material

Supplementary material
