# Supplementary material for: Efficacy of an indicated prevention strategy on sickness absence and termination of the employment contract: a 5-year follow-up study
Source: Scand J Work Environ Health. 2021 Apr 27;47(4):258–67. doi: 10.5271/sjweh.3945 (PMC8091073; doi:10.5271/sjweh.3945)
Supplement: Supplementary material [file SJWEH-47-258-S001.pdf]

Efficacy of an indicated prevention strategy on sickness absence and termination of the employment contract: a 5-year follow-up study[1]

by Sophie H Klasen, MSc,[2] Ludovic GPM van Amelsvoort, PhD, Nicole WH Jansen, PhD, Jos JM Slangen, Gladys Tjin A Ton, MD, IJmert Kant, PhD

1. *Supplementary material*
2. *Correspondence to: Sophie H Klasen, CAPHRI School for Public Health and Primary Care. Department of Epidemiology, Faculty of Health, Medicine and Life Sciences, Maastricht University, Maastricht*, The Netherlands. [E-mail: sophie.klasen@maastrichtuniversity.nl]

Screening questionnaire

The questionnaire entailed 34 multiple choice questions regarding demographics, work environment, private situation, (mental) health, and SA history. The screening instrument was used to predict which employees were at risk for SA, this resulted for RCT I in a specificity of the Balansmeter of 94.4% and a sensitivity of 49.4%. For women, a cut-off point with 94.7% specificity and 30.3% sensitivity was applied (16). For RCT II, the specificity for men was 87.8% and for women 87.9% with a sensitivity for men of 65.1% and for women 52.6% (17).

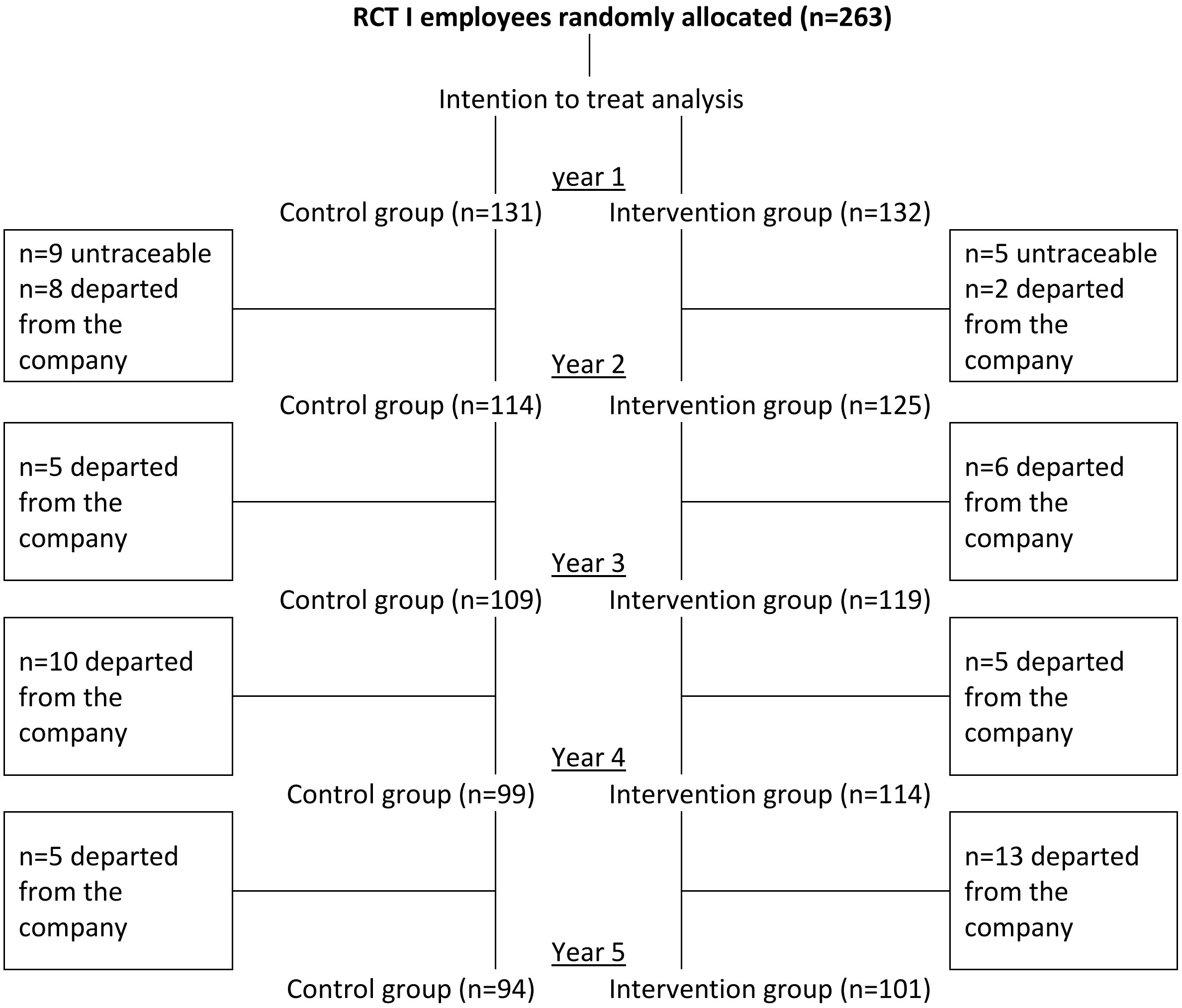

Flow diagram S1 of RCT I participant allocation and departure from the company

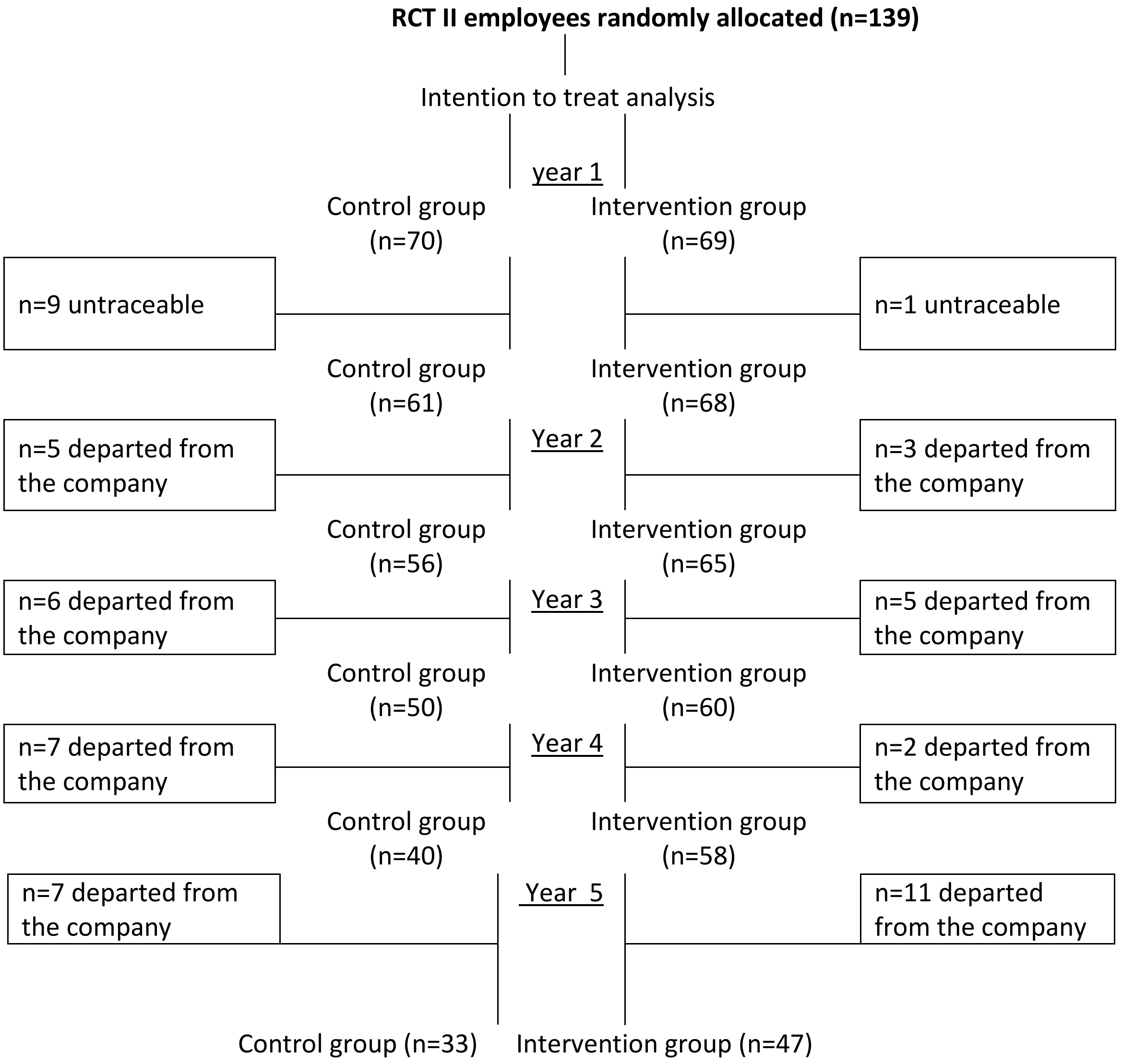

Flow diagram S2 of RCT II participant allocation and departure from the company

Table S1: Overview efficacy of the indicated prevention strategy on SA parameters for RCT I (per protocol analysis)

2-years follow-up	Control group			Intervention group			Mean difference	P-value [1]	P-value [2]
	Mean/ (SD)	Median	n	Mean/ (SD)	Median	n			
Total SA duration±	68.4 (88.0)	35.8	114	43.9 (62.0)	20.0	95	24.5	0.008	0.002
SA frequency	5.42 (5.47)	4.0	114	4.50 (3.45)	4.0	95	0.92	0.114	0.020
Percentage of LTSA*	26.7%		35	21.2%		21	5.5%	0.157	0.115
**3-years follow-up**									
Total SA duration±	104.3 (119.8)	56.6	109	75.4 (105.2)	32.0	92	28.8	0.043	0.026
SA frequency	7.66 (7.38)	6.0	109	6.38 (4.89)	5.0	92	1.28	0.111	0.015
Percentage of LTSA*	36.6%		48	23.2%		23	13.4%	0.008	0.004
**4-years follow-up**									
Total SA duration±	125.2 (150.4)	65.0	99	105.1(156.0)	46.0	87	20.1	0.301	0.223
SA frequency	9.36 (9.43)	7.0	99	8.06 (6.02)	7.0	87	1.3	0.211	0.030
Percentage of LTSA*	35.1%		46	25.3%		25	9.8%	0.023	0.017
**5-years follow-up**									
Total SA duration±	166.3 (202.7)	93.5	94	113.5 (175.2)	58.4	78	52.8	0.034	0.035
SA frequency	11.44 (11.18)	9.0	94	9.91 (7.14)	8.0	78	1.53	0.245	0.059
Percentage of LTSA*	35.9%		47	22.2%		22	13.7%	0.009	0.004

[1] Crude analysis using Poisson regression without adjustments for covariates. [2]Adjusted analysis using Poisson regression for covariates; age, gender, job function and long-term illness. ±*Total SA duration including >28 days SA *Percentage LTSA is calculated annually*

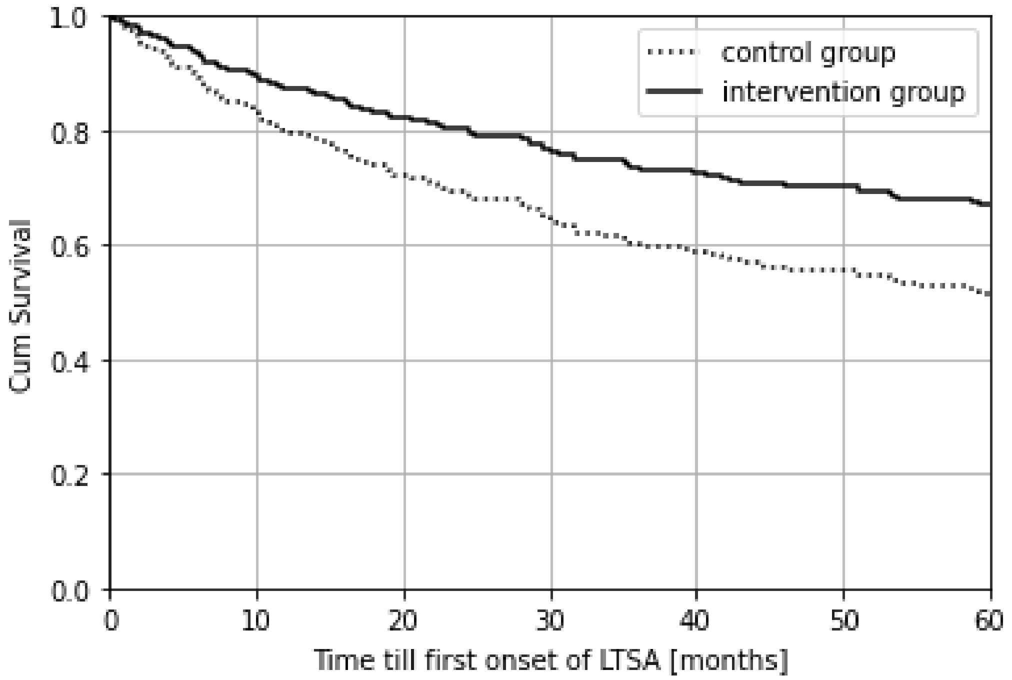

Figure S1: Time till first onset of LTSA in months (RCT I per protocol analysis) (HR 0.53; 0.34-0.83)

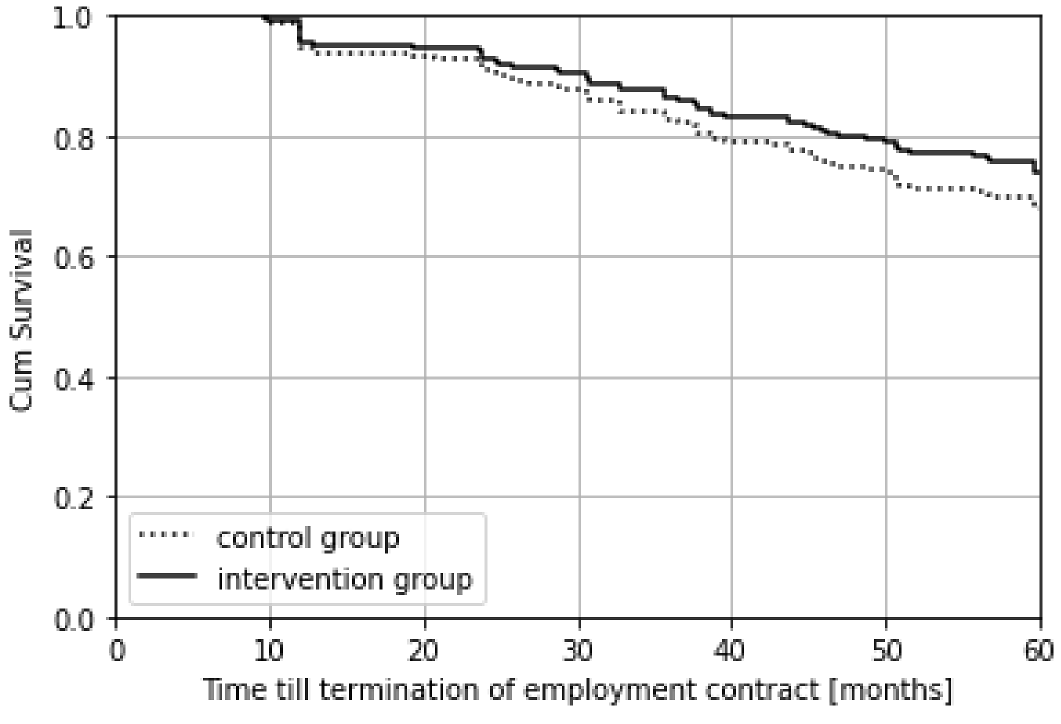

Figure S2: Time till termination of the employment contract with the company in months (RCT I per protocol analysis) (HR 0.78; 0.47-1.30).

Table S2: Overview efficacy of the indicated prevention strategy on SA parameters for RCT II (per protocol analysis)

2-years follow-up	Control group			Intervention group			Mean difference	P-value [1]	P-value [2]
	**Mean/ (SD)**	**Median**	**n**	**Mean/ (SD)**	**Median**	**n**			
Total SA duration±	81.2 (114.2)	29.5	56	48.8 (70.5)	22.0	43	32.3	0.056	0.079
SA frequency	3.68 (2.88)	3.0	56	3.12 (2.46)	3.0	43	0.56	0.300	0.343
Percentage of LTSA*	18.6%		13	19.6%		9	-1%	0.772	0.754
**3-years follow-up**									
Total SA duration±	106.6 (135.8)	43.5	50	93.5 (119.3)	34.0	40	13.1	0.595	0.560
SA frequency	5.46 (4.04)	4.5	50	4.62 (3.53)	4.0	40	0.84	0.284	0.220
Percentage of LTSA*	21.4%		15	32.6%		15	-11.2%	0.479	0.538
**4-years follow-up**									
Total SA duration±	134.0 (185.2)	36.5	40	124.7 (179.8)	35.0	39	9.3	0.796	0.688
SA frequency	6.45 (5.42)	5.0	40	5.92 (4.05)	5.0	39	0.53	0.599	0.591
Percentage of LTSA*	17.1%		12	30.4%		14	-13.3%	0.598	0.747
**5-years follow-up**									
Total SA duration±	170.5 (249.0)	54.1	33	184.8 (251.4)	91.0	33	-14.3	0.794	0.869
SA frequency	7.15 (5.97)	6.0	33	7.03 (5.06)	6.0	33	0.12	0.923	0.869
Percentage of LTSA*	17.1%		12	30.4%		14	-13.3%	0.652	0.748

[1] Crude analysis using Poisson regression without adjustments for covariates. [2]Adjusted analysis using Poisson regression for covariates; age, gender, education level and long-term illness. . ±*Total SA duration including >28 days SA *Percentage LTSA is calculated annually*

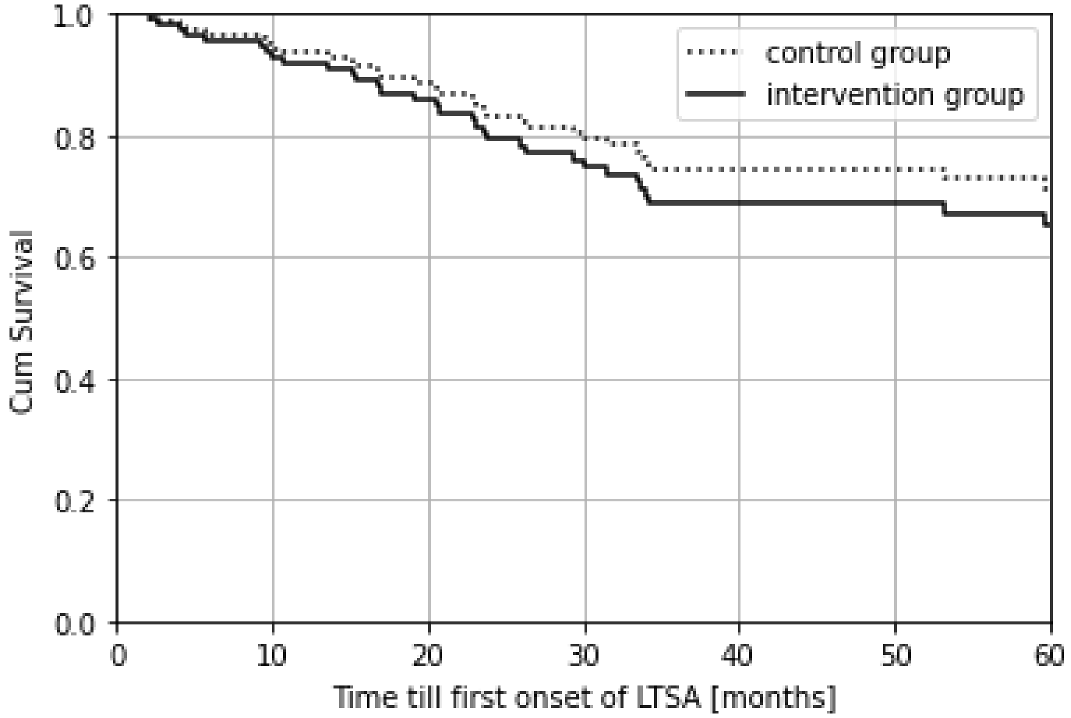

Figure S3: Time till first spell of LTSA (RCT II per protocol analysis) (HR 1.27; 0.63-2.56)

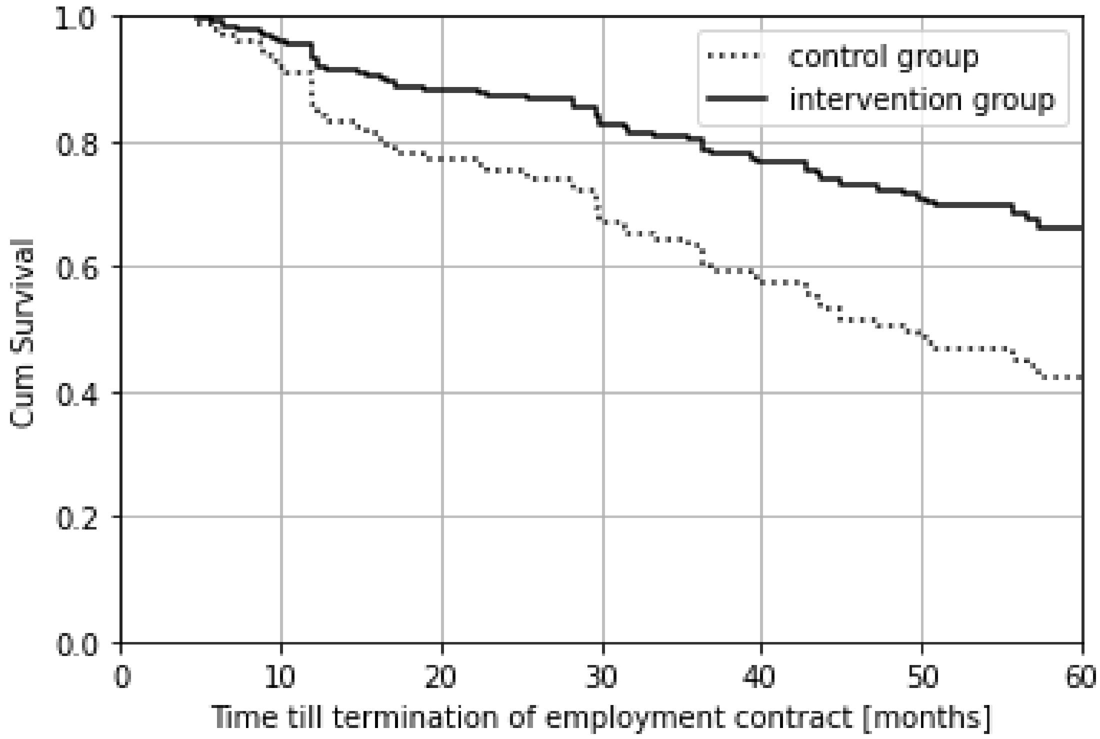

Figure S4: Time till termination of the employment contract with the company in months (RCT II per protocol analysis) (HR 0.48; 0.27-0.85)
